# Long term opioid use after burn injury: a retrospective cohort study

**DOI:** 10.1016/j.bja.2023.12.003

**Published:** 2024-01-11

**Authors:** Sherzah Jamal, Martin Shaw, Tara Quasim, Kathryn Puxty, Christopher McGovern

**Affiliations:** 1 School of Medicine, Dentistry and Nursing, University of Glasgow, Glasgow, UK; 2 Department of Intensive Care, Glasgow Royal Infirmary, Glasgow, UK

**Keywords:** burn injury, opioid dependence, opioids, pancreatitis, postoperative pain

## Abstract

**Background:**

Patients who have survive a burn injury might be at risk of opioid dependence after discharge. This study examined the use of opioids in patients who suffer burn injury and explored factors associated with persistent opioid use after hospital discharge.

**Methods:**

This retrospective cohort study compared adults admitted with a burn injury from 2009 to 2019 with two matched comparison cohorts from the general population and adults with a diagnosis of acute pancreatitis. Pre-admission prescription opioid use was determined, and a multivariable negative binomial regression analysis used to explore post-discharge opioid use.

**Results:**

A total of 7147 burn patients were matched with 6810 pancreatitis patients and with 28 184 individuals from the general population. Pre-admission opioid use was higher in the burn and pancreatitis cohorts (29% and 40%, respectively) compared with the general population (17%). Opioid use increased in both burn and pancreatitis cohorts after discharge (41% and 53%, respectively), although patients with pancreatitis were at even higher risk of increased opioid use in an adjusted analysis (incidence rate ratio 1.43). Female sex, lower socioeconomic status, ICU admission, pre-injury opioid use, and a history of excess alcohol use were all associated with an increase in opioid prescriptions after discharge.

**Conclusions:**

Opioid use is high in those admitted with a burn injury or acute pancreatitis when compared with the general population, increasing further after hospital discharge. Female sex and socioeconomic deprivation are among factors that make increased opioid use more likely, although this phenomenon seems even more pronounced in those with acute pancreatitis compared with burn injuries.

**Editor's key points**

• Burns cause 180,000 deaths per year worldwide and can not only provoke excruciating acute pain but also chronic pain, which is generally treated with opioids.
• In this large retrospective cohort survey conducted in Scotland, use of opioids in patients admitted to hospital for a burn injury was compared to two matched control groups: patients with acute pancreatitis and subjects from the general population.
• The authors found that 41% of patients with burn injury received an opioid in the year following discharge. This was predicted by female sex, socioeconomic deprivation, ICU admission, prior opioid use and excess alcohol use. A similar trend was observed after acute pancreatitis.
• This study emphasizes the need to monitor discharge prescriptions of opioids in patients with burn injury, especially given the common risk factors seen between burn injury and opioid dependency.

Worldwide, burns are the cause of 180,000 deaths per year, most of which are in low-income countries.^1^ Risk factors for burns include overcrowding, poor safety practice, and low socioeconomic status.^2^ In Scotland, around 500 patients are admitted to hospital each year, of whom 5% are major burn injuries.^3^^,^^4^

Pain from a burn injury can be excruciating. This is usually attributed to the damage caused by the burn but can also be attributable to associated injuries and interventions such as skin grafting. Poor pain control can prolong the healing process by causing stress to the patient resulting in release of cortisol and catecholamines. This can lead to a longer hospital stay which comes with its own physical and psychological burdens.^5^ Chronic pain is common after burn injury and can have a significant impact on quality of life,^6^ commonly being associated with higher levels of anxiety and depression.^7^

Opioids are the mainstay of analgesia for major burns.^5^^,^^8^, ^9^, ^10^ Although they are effective drugs for pain management, their side-effect profile is far from benign including unwanted effects such as nausea, respiratory depression, and delirium. Perhaps the most notable consequence of opioid use is the potential for dependence.^6^^,^^11^ It has been suggested that burn patients are more likely to develop opioid dependence after discharge.^8^^,^^9^ This can be because of large doses and long-acting opioids given during hospital stay. Risk factors commonly associated with opioid misuse such as mental health conditions, other substance dependence, previous opioid use, and male sex^11^ are also recognised risk factors for sustaining a burn injury.^7^

Previous research has shown that patients who sustain even minor burn injuries are at a higher risk of developing opioid dependence compared with other surgical populations.^12^ Prolonged opioid use poses a big challenge with a 75% increase in hospital stays attributable to opioids being seen in the USA.^11^^,^^13^ It has been suggested that Scotland has an even greater opioid crisis than the USA and the rest of the UK,^14^ with opioid-related drug deaths having more than doubled from 2009 to 2018.^15^ Although it was previously believed that there was little evidence for developing an opioid dependence after a burn injury,^8^ recent studies have shown that there may be a link.^6^^,^^13^^,^^16^ There are conflicting results among these studies regarding what factors may lead to an increased likelihood of opioid dependence.

Another painful condition that can result in similar local and systemic changes with the potential for multi-organ dysfunction is acute pancreatitis, a condition previously described as a ‘burn-like injury’ in the retroperitoneal space.^17^, ^18^, ^19^, ^20^, ^21^ In Scotland, gallstones and alcohol excess are the most common causes of pancreatitis, with the condition often affecting people from areas of socioeconomic deprivation.^22^^,^^23^ Pain is very common in acute pancreatitis, with opioids being the main analgesic administered.^20^ These considerations make patients with acute pancreatitis a suitable comparator for our study.

The aim of this study was to describe the pre- and post-admission use of opioids in patients admitted to hospital with a burn injury, comparing them with matched cohorts from the general population and those admitted with a diagnosis of acute pancreatitis and to explore the difference between and factors associated with persisting opioid use in the burn and pancreatitis cohorts after hospital discharge.

## Methods

This study was carried out as a retrospective cohort study using national administrative data sources including the National Records Scotland (NRS), Prescription Information System (PIS), Care of Burns in Scotland (COBIS), and records of acute (SMR01) and psychiatric (SMR04) hospital admissions.

### Inclusion criteria

All adults >16 yr old admitted to hospital in Scotland with a primary diagnosis of burn injury from January 1, 2009 to December 31, 2019 were included. Two additional cohorts were then matched; firstly, those with an acute hospital admission with a diagnosis of acute pancreatitis and secondly a cohort of individuals selected from the general population from the Community Health Index (CHI) database without either of the aforementioned diagnoses.

Patients from the acute pancreatitis cohort were matched to the index burn-injured cohort in a 1:1 ratio, matched on sex, socioeconomic deprivation, and age. The general population cohort were matched using the same criteria in a 1:4 ratio. Patients in all cohorts were >16 yr old.

### Data

Patient demographic data were extracted from SMR01 using the Scottish Index of Multiple Deprivation (SIMD) to describe socioeconomic deprivation. The SIMD is the Scottish Government's standard approach to identify areas of deprivation taking into account factors such as income, employment, education, health, access to services, crime, and housing.^24^ Quintile 1 translates to the 20% most deprived in Scotland whilst quintile 5 to the 20% least deprived in Scotland. Burn injury details were extracted from both the COBIS and SMR01 data. Ethnicity data was gathered from SMR01 or the CHI database and simplified from >20 subcategories into five broad categories. Comorbidity data were extracted from the COBIS, SMR01, and SMR04 datasets in the 5 yr preceding the index hospital admission using relevant ICD-10 (International Statistical Classification of Diseases and Related Health Problems) codes. These data were converted to classify conditions using the Elixhauser comorbidity index.^25^ This was used to form total morbidity which was a count of the number of different conditions the patient had. If an individual had two or more conditions, they were classified as multimorbid.

Drug prescription data for 1 yr pre-admission and 1 yr after discharge were extracted from the PIS, a database of all NHS Scotland prescriptions dispensed in the community. For those in the general population cohort, the index date of admission was taken to be the corresponding admission date of the individual they were matched to in the burn cohort.

### Approval

Datasets were linked by the electronic Data Research and Innovation Service (eDRIS), part of Public Health Scotland (PHS). Proportionate governance approval was granted by the Public Benefit and Privacy Panel (PBPP), reference: 1819-0287. Research ethics approval (REC) was granted by the NHS Health Research Authority, REC reference: 20/HRA/1590, IRAS project ID: 263159.

### Outcomes

The primary outcome was the number of patients prescribed an opioid in the 12 months after discharge from hospital. Secondary outcomes were the number of recurrent users of opioids (classified as receiving three or more prescriptions) and the number of opioid prescriptions dispensed.

### Statistical analysis

Statistical analyses were performed using R version 3.6.1 (Foundation for Statistical Computing, Vienna, Austria). Analysis of the differences in comorbidities between each cohort were carried out using Pearson's χ^2^ test or Fisher's exact test as appropriate. To explore factors associated with the number of opioid prescriptions dispensed after discharge, only patients from the burns and pancreatitis cohorts were included. Patients who died during hospital admission were excluded and a negative binomial regression analysis used to analyse variables associated with the number of opioid prescriptions in the year after discharge. Univariable analysis was conducted first to assess any variables associated with the number of opioid prescriptions after discharge. Any variables with a *P*-value <0.1 were included in the multivariable analysis and a backwards stepwise regression approach used to build a model. Statistical significance was set at a *P*-value <0.05.

## Results

**Fig 1:**
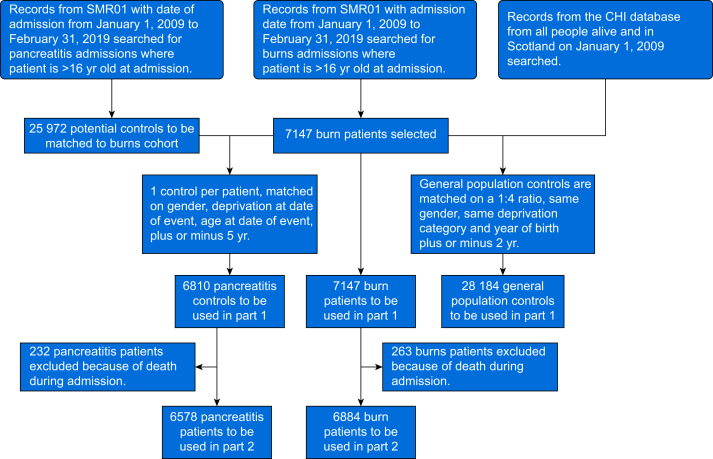
Consort diagram illustrating the synthesis of each cohort. CHI, Community Health Index. SMR01, Scottish Morbidity Record of general/ acute inpatient and day case.

During the study period 7147 burn patients were identified. These patients were matched with 6810 patients from the pancreatitis cohort and 28 184 patients from the general population. Details of the matching criteria can be seen in Figure 1. Patients across the three cohorts were well matched for baseline patient characteristics including age, sex, and SIMD as seen in Table 1.

**Table 1:** Baseline patient characteristics in each cohort. IQR, inter-quartile range. NA, not applicable.

Characteristic	Burn, *N*=7147	General population, *N*=28 184	Pancreatitis, *N*=6810
Age (yr), median (IQR)	47 (32–64)	48 (32–65)	49 (35–66)
Sex, *n* (%)			
Male	4556 (64)	17 904 (64)	4240 (62)
Female	2591 (36)	10 280 (36)	2570 (38)
Scottish Index of Multiple Deprivation, *n* (%)			
1	2116 (30)	8107 (29)	2070 (30)
2	1667 (23)	6345 (23)	1585 (23)
3	1335 (19)	5444 (19)	1343 (20)
4	1127 (16)	4605 (16)	1010 (15)
5	792 (11)	3516 (12)	792 (12)
Unknown	110 (1.5)	167 (0.6)	10 (0.1)
ICU Admission, *n* (%)	590 (8.3)	NA	428 (6.3)
Ethnicity, *n* (%)			
Any White background	5635 (79)	11 491 (41)	5752 (84)
Any mixed ethnic group	11 (0.2)	26 (0.1)	11 (0.2)
Any Asian background	77 (1.1)	145 (0.5)	76 (1.1)
Other ethnic group	45 (0.6)	52 (0.2)	22 (0.3)
Any Black background	18 (0.3)	36 (0.1)	24 (0.4)
Unknown ethnicity	1361 (19)	16 434 (58)	925 (14)
Comorbidity, *n* (%)			
No comorbidity	5797 (81)	26 178 (93)	5608 (82)
One comorbidity	993 (14)	1646 (5.8)	921 (14)
Two or more comorbidities	357 (5.0)	360 (1.3)	281 (4.1)
Alcohol abuse	403 (5.6)	149 (0.5)	466 (6.8)
Drug abuse	85 (1.2)	26 (<0.1)	23 (0.3)
Psychoses	70 (1.0)	59 (0.2)	21 (0.3)
Depression	90 (1.3)	68 (0.2)	40 (0.6)
Prescription drug use, *n* (%)			
Antipsychotics	453 (6.3)	535 (1.9)	287 (4.2)
Antiepileptics	894 (13)	1155 (4.1)	580 (8.5)
Antidepressants	2110 (30)	3943 (14)	1990 (29)
Anxiolytics	1301 (18)	1963 (7.0)	1181 (17)
Drugs for substance dependence	676 (9.5)	905 (3.2)	514 (7.5)

Patients with a burn injury or acute pancreatitis had a significantly higher comorbidity burden than those in the general population cohort across multiple categories (Table 1 and Supplementary material). Alcohol abuse was the most common comorbidity in both the burn cohort (5.6%) and pancreatitis cohort (6.8%), both significantly higher than the general population (0.5%). Drug abuse, depression, and psychosis were also higher in the burn cohort (*P*-value <0.001). The use of prescription drugs across multiple categories was also significantly higher in both the burn and pancreatitis cohorts compared with the general population, especially regarding drugs used for mental health conditions or substance dependence (Table 1 and Supplementary material).

Only 2276 (32%) patients in the burn cohort had data on the mechanism of injury and 3012 (42%) on the size of burn (see Supplementary material). From available data, hot liquid burns were the most common burn type (34%) and the majority of burns affected <20% total body surface area (TBSA) (94%). Burns affecting 20–49% of TBSA accounted for 3.9% of injuries and only 2.3% had a burn >50% TBSA. Only 1.6% of patients had a known airway burn and 1.9% had a known associated smoke inhalation injury.

Pre-admission opioid prescriptions were higher in both the burns cohort (29%) and pancreatitis cohort (40%) than the general population (17%) (Table 2). The most common opioid prescribed was co-codamol across all three cohorts.

**Table 2:** Pre-injury opioid use and post-discharge opioid use of patients in each cohort. The numbers of each type of drug prescribed show the number of patients receiving that drug, with each patient potentially receiving more than one opioid drug. NA, not applicable. ∗*n* (%) or mean (minimum) (maximum).

Characteristic	Pre-injury opioid use			Post-discharge opioid use	
	Burn, *N*=7147∗	General population, *N*=28 184∗	Pancreatitis, *N*=6810∗	Burn, *N*=6884∗	Pancreatitis, *N*=6578∗
Receiving opioid prescriptions	2057 (29)	4888 (17)	2735 (40)	2805 (41)	3464 (53)
Recurrent opioid user (≥3 prescriptions)	1276 (18)	2675 (9.5)	1613 (24)	1546 (22)	2141 (33)
Number of opioid prescriptions	2.0 (0.0) (66.0)	1.0 (0.0) (68.0)	2.6 (0.0) (100.0)	2.5 (0.0) (81.0)	3.9 (0.0) (117.0)
Opioid-naive pre-admission	5090 (71)	23 296 (83)	4075 (60)	NA	NA
Opioid-naive patient who became an opioid user after discharge	NA	NA	NA	1371 (20)	1459 (22)
Opioid-naive patient who became recurrent user after discharge	NA	NA	NA	480 (6.9)	613 (9.3)
Co-codamol	1227 (17)	3227 (11)	1674 (25)	1395 (20)	1700 (26)
Tramadol hydrochloride	498 (7.0)	1019 (3.6)	686 (10)	705 (10)	1249 (19)
Fentanyl	44 (0.6)	57 (0.2)	42 (0.6)	49 (0.7)	77 (1.2)
Co-dydramol	185 (2.6)	538 (1.9)	279 (4.1)	174 (2.5)	262 (4.0)
Dihydrocodeine tartrate	303 (4.2)	448 (1.6)	366 (5.4)	672 (9.8)	703 (11)
Morphine	303 (4.2)	448 (1.6)	366 (5.4)	318 (4.6)	484 (7.4)
Oxycodone	75 (1.0)	105 (0.4)	70 (1.0)	138 (2.0)	207 (3.1)
Codeine phosphate	127 (1.8)	198 (0.7)	178 (2.6)	229 (3.3)	262 (4.0)
Co-codamol with buclizine hydrochloride	27 (0.4)	39 (0.1)	21 (0.3)	16 (0.2)	14 (0.2)
Buprenorphine	19 (0.3)	54 (0.2)	26 (0.4)	35 (0.5)	32 (0.5)
Other opioid	21 (0.3)	57 (0.2)	33 (0.5)	45 (0.7)	55 (0.8)

Exploring post-discharge opioid prescriptions, 263 burn-injured patients and 232 patients with pancreatitis were excluded as they died during hospital admission. Therefore 6884 burns patients and 6573 pancreatitis patients were included in the post-injury opioid use analysis (Fig. 1). The number of patients being prescribed opioids post-discharge increased by >10% in both the burns cohort (41%) and pancreatitis cohort (53%), as did the number and proportion of patients defined as recurrent users, receiving three or more opioid prescriptions (Table 2). Of note, 480 (6.9%) of the patients in the burn cohort and 613 (9.3%) in the pancreatitis cohort who received three or more opioid prescriptions after discharge were opioid naive in the 12 months preceding hospital admission. The mean number of prescriptions dispensed to patients also increased in both cohorts.

**Fig 2:**
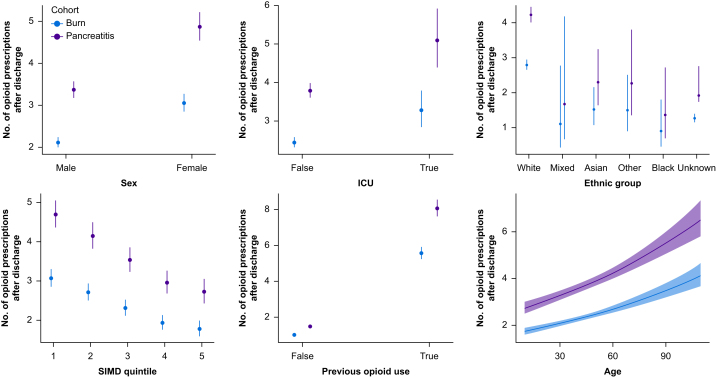
Graphical representation of number of opioid prescriptions after discharge when comparing different factors of interest using univariable analysis of both cohorts. SIMD, Scottish Index of Multiple Deprivation.

Univariable analysis of factors associated with the number of opioid prescriptions after discharge is seen in Table 3. The pancreatitis cohort were at a higher risk of increased opioid use after their injury compared with the burn cohort (IRR 1.55, confidence interval [CI] 1.44–1.66, *P*<0.001). Females were more likely to be prescribed more opioids than males (IRR 1.45, CI 1.34–1.56, *P*<0.001) (Fig. 2). Previous opioid use, multimorbidity, admission to ICU, and socioeconomic deprivation (Fig. 2) were all associated with higher opioid prescriptions after discharge. Increasing severity of burn injury, as measured by %TBSA, was also associated with higher opioid use in an analysis excluding those with pancreatitis.

**Table 3:** Univariable analysis of factors associated with an increase in opioid prescriptions after discharge from index admission. In all factors the cohort was accounted for when performing statistical tests. CI, confidence interval; IRR, incidence rate ratio; SIMD, Scottish Index of Multiple Deprivation; TBSA, total body surface area. ∗Calculated for each 1-yr increase in age. ^†^Calculated for the burns cohort only.

Factor	IRR	95% CI	*P*-value
Cohort			
Burn	Reference	Reference	
Pancreatitis	1.55	1.44–1.66	<0.001
Age (yr)∗	1.01	1.01–1.01	<0.001
Sex			
Male	Reference	Reference	
Female	1.45	1.34–1.56	<0.001
Ethnic group			
Any White background	Reference	Reference	
Any mixed ethnic group	0.4	0.17–1.11	0.048
Any Asian background	0.54	0.39–0.78	<0.001
Other ethnic group	0.54	0.33–0.94	0.019
Any Black background	0.32	0.17–0.68	0.001
Unknown ethnicity	0.45	0.41–0.50	<0.001
SIMD quintile			
1	Reference	Reference	
2	0.88	0.80–0.97	0.012
3	0.75	0.68–0.83	<0.001
4	0.63	0.56–0.70	<0.001
5	0.58	0.51–0.66	<0.001
Pre-injury opioid use	5.42	5.08–5.79	<0.001
ICU	1.34	1.16–1.56	<0.001
Total morbidity count	1.39	1.30–1.49	<0.001
Multimorbidity	1.58	1.33–1.89	<0.001
Alcohol abuse	1.43	1.24–1.67	<0.001
Drug abuse	1.05	0.72–1.62	0.8
Depression	1.51	1.06–2.24	0.031
Psychoses	1.13	0.75–1.81	0.6
TBSA^†^			
<20%	Reference	Reference	
20–49%	2.39	1.49–4.13	<0.001
>50%	2.22	1.13–5.21	0.038

Multivariable analysis of both cohorts is shown in Table 4. Female sex, lower socioeconomic status, ICU admission during hospital stay, pre-injury opioid use, and a history of alcohol excess were all associated with an increase in opioid prescriptions after discharge. Patients from some ethnic minority backgrounds were less likely to have opioid prescriptions after discharge compared with white participants. People from more affluent areas of residence were also less likely to be prescribed opioids after discharge compared with those from more deprived areas.

**Table 4:** Multivariable analysis of factors associated with an increase in opioid prescriptions after discharge from index injury. CI, confidence interval; IRR, incidence rate ratio; SIMD, Scottish Index of Multiple Deprivation.

Factor	IRR	95% CI	*P*-value
Cohort			
Burn	Reference	Reference	
Pancreatitis	1.43	1.34–1.53	<0.001
Sex			
Male	Reference	Reference	
Female	1.15	1.07–1.23	<0.001
Ethnic group			
Any White background	Reference	Reference	
Any mixed ethnic group	0.49	0.22–1.20	0.091
Any Asian background	0.65	0.48–0.90	0.008
Other ethnic group	0.66	0.42–1.10	0.093
Any Black background	0.53	0.29–1.02	0.043
Unknown ethnicity	0.66	0.60–0.72	<0.001
SIMD quintile			
1	Reference	Reference	
2	0.93	0.85–1.02	0.11
3	0.84	0.77–0.92	<0.001
4	0.74	0.67–0.82	<0.001
5	0.74	0.66–0.83	<0.001
ICU	1.7	1.49–1.94	<0.001
Pre-injury opioid use	5.06	4.73–5.41	<0.001
Alcohol abuse	1.2	1.06–1.37	0.006

In a separate multivariable analysis including only those with a burn injury, increasing size of burn injury was associated with increased opioid prescriptions in those with a burn 20–49% TBSA (IRR 3.18, 95% CI 2.05–5.15, *P*<0.001) but the correlation in burns >50% TBSA did not reach statistical significance (IRR 1.77, CI 0.98–3.57, *P*=0.078) (see Supplementary material).

## Discussion

This study has explored the use of opioid prescriptions in a cohort of burn-injured patients compared with two control groups. The pre-injury use of these drugs was significantly higher in those who suffered a burn injury compared with the general population. Comorbidities including depression, psychosis, and drug abuse were also found to be more prevalent among this cohort, echoing similar results found in other studies.^2^

After burn injury, 41% of patients received an opioid prescription in the year after discharge. This is higher than results seen in other studies where 25% of patients received an opioid prescription 7 days after discharge^11^ or 30% were prescribed opioids on day 30 after discharge.^6^ Both of these studies were conducted in the USA; to our knowledge this is the first study to be conducted in the UK.

Although males accounted for a higher proportion of each cohort, females were more likely to receive opioid prescriptions after discharge. This is in contrast to previous studies that have not demonstrated an association between sex and opioid use after burn injury.^6^^,^^16^ However, studies exploring opioid use after other conditions, including pancreatitis and spinal cord dysfunction, have found a positive association between female sex and increased opioid use.^26^^,^^27^

Socioeconomic deprivation has consistently been demonstrated to be a risk factor for a multitude of health conditions, including sustaining a burn injury, and other health outcomes. This has been reflected in this study with patients from more affluent geographical areas being at less risk of being prescribed opioids after burn injury. This phenomenon linking deprivation and opioid use has been demonstrated in other studies.^28^

Admission to the ICU was associated with higher opioid use after discharge in this study in both univariable and multivariable analysis. The association between ICU admission and opioid dependency has been debated over the past decade, with research finding conflicting results.^29^^,^^30^ However, with a recognition that there is a frequent requirement for opioid analgesia in the critical care setting, often regardless of the underlying pathology, and a high prevalence of chronic pain after ICU survival, there has been growing interest in measures to reduce the risk of both chronic pain and the risk of opioid dependency after ICU discharge.^29^

Individuals from Asian or Black ethnic backgrounds were found to be at a lower risk of opioid prescriptions after discharge compared with people from a White ethnic background. Deficiencies in the use of analgesic medications for people from ethnic minorities have been highlighted in recent studies involving various pathologies or conditions.^31^^,^^32^ The results of this study might reflect this phenomenon but should be interpreted with caution given the low number of patients of ethnic minority background and significant number of individuals with missing data regarding ethnicity.

As opioid use increased after burn injury, a similar trend was observed in those with acute pancreatitis, reflecting the findings of other studies.^33^^,^^34^ Opioid use was higher in the pancreatitis cohort compared with those with a burn injury in both pre-admission and post-discharge analysis. This difference between cohorts persisted in the multivariable binomial regression analysis, perhaps accounted for by the higher prevalence of opioid use pre-admission in the pancreatitis cohort or persisting pain because of recurrent or chronic pancreatitis.^35^ Pre-existing opioid use was associated with increased post-discharge prescriptions, reflecting previous studies that demonstrate prior opioid use being a risk factor for developing chronic opioid use.^11^^,^^26^

### Strengths and limitations

To our knowledge this is the first study comparing opioid prescribing patterns in patients who suffer a burn injury in comparison with two matched cohorts. The large patient groups with well-matched controls allows for appropriate reflection of results across the population. The datasets used in this study are national databases that are well integrated within Scotland's healthcare system and undergo regular audit to ensure validity of the data.^36^ Exploratory analysis of factors associated with the number of opioid prescriptions using multivariable binomial regression analysis allowed better interpretation of post-discharge opioid prescriptions given the wide variation in the number of prescriptions each individual received.

This study has several limitations. Firstly, the use of administrative healthcare databases are recognised to lack the granularity of data to fully describe cohorts, especially regarding elements such as pre-existing comorbidity.^37^ By using the number of opioid prescriptions in the 12 months after discharge this did not account for the chronological relationship between the index event and the opioid prescriptions, perhaps detecting additional opioid prescriptions given for other conditions. Additionally, this study does not accurately quantify the incidence of pain after burn injury, rather uses opioids as a surrogate marker of pain. Furthermore, although this study shows an increase in number of prescriptions after discharge, it is limited in making any conclusions regarding the appropriateness of these prescriptions or any harm associated with them.

### Conclusions

Individuals who have been hospitalised with a burn injury are more likely to be prescribed opioids, have mental health conditions, and have substance abuse problems compared with patients of the same sex, age, and socioeconomic deprivation from the general population. Risk factors for increased opioid use after discharge after burn injury include female sex, socioeconomic deprivation, ICU admission, pre-injury opioid use, and a history of alcohol abuse. In comparison to the similar painful, inflammatory, multisystem condition of acute pancreatitis, burn-injured patients were not at any additional risk of increased opioid use after discharge. This might be explained by the high prevalence of opioid use in patients with acute pancreatitis, the high burden of comorbidity in this comparator cohort, or the likelihood of developing recurrent or chronic pancreatitis or even pancreatic cancer.

Opioids are associated with dependence and harm. This study helps to emphasise the vigilance required when prescribing opioids in this patient group, especially given the abundance of common risk factors seen in burn injury and opioid dependency such as alcohol abuse and mental health conditions.

## Authors’ contributions

Study conception and design: CM, KP, TQ

Data acquisition: CM, TQ

Data analysis: CM, SJ, MS

Data interpretation: CM, SJ, MS, KP, TQ

Drafting of the manuscript: SJ, CM

Revising of manuscript critically for important intellectual content: CM, SJ, MS, KP, TQ

Final approval of manuscript: all authors

All authors agree to be accountable for all aspects of this work.

## Declaration of interest

The authors declare that they have no conflicts of interest.

## Funding

UK National Institute of Academic Anaesthesia (NIAA) Association of Anaesthetists research grant (NIAA19R213); NHS Greater Glasgow and Clyde Endowment Fund (GN19AE535); and National Institute of Academic Anaesthesia (NIAA) Association of Anaesthetists John Snow Anaesthesia Intercalated Award.
