# Supplementary material for: Long term opioid use after burn injury: a retrospective cohort study
Source: Br J Anaesth. 2024 Jan 11;132(3):599–606. doi: 10.1016/j.bja.2023.12.003 (PMC10870133; doi:10.1016/j.bja.2023.12.003)
Supplement: Multimedia component 1 [file mmc1.docx]

Long term opioid use following a burn injury:

Supplementary Material

Contents

1. Supplementary Table 1: Number of patients in each cohort prescribed each drug one year before injury.
2. Supplementary Table 2: Description of burn sustained by patients in the burns cohort.
3. Supplementary Table 3- Univariable analysis of factors associated with an increase in opioid prescriptions following discharge from burn admission.
4. Supplementary Figure 1- Graphical representation of number of opioid prescriptions post discharge when comparing different factors of interest using univariable analysis of both cohorts.
5. Supplementary Table 4- Subgroup multivariable analysis of factors associated with an increase in opioid prescriptions following discharge from a burn injury. This table includes just the burns cohort.

| **Drug** | Burn,  N = 7,147* | General Population,  N = 28,184* | Pancreatitis,  N = 6,810* | p-value† |
| --- | --- | --- | --- | --- |
| Gabapentinoids | 512 (7.2%) | 723 (2.6%) | 382 (5.6%) | <0.001 |
| Opioids | 2,057 (29%) | 4,888 (17%) | 2,735 (40%) | <0.001 |
| Antipsychotics | 453 (6.3%) | 535 (1.9%) | 287 (4.2%) | <0.001 |
| Antiepileptics | 894 (13%) | 1,155 (4.1%) | 580 (8.5%) | <0.001 |
| Antidepressants | 2,110 (30%) | 3,943 (14%) | 1,990 (29%) | <0.001 |
| Anxiolytics | 1,301 (18%) | 1,963 (7.0%) | 1,181 (17%) | <0.001 |
| Analgesia | 2,644 (37%) | 6,431 (23%) | 3,283 (48%) | <0.001 |
| Drugs for substance dependence | 676 (9.5%) | 905 (3.2%) | 514 (7.5%) | <0.001 |
| Cardiovascular drugs | 2,504 (35%) | 7,363 (26%) | 2,921 (43%) | <0.001 |
| Gastrointestinal drugs | 2,673 (37%) | 7,109 (25%) | 4,210 (62%) | <0.001 |
| MSK drugs | 1,780 (25%) | 5,119 (18%) | 1,903 (28%) | <0.001 |
| Endocrine drugs | 1,446 (20%) | 4,000 (14%) | 1,616 (24%) | <0.001 |
| Respiratory drugs | 1,685 (24%) | 4,568 (16%) | 1,768 (26%) | <0.001 |
| Nutrition and blood drugs | 1,689 (24%) | 3,098 (11%) | 1,920 (28%) | <0.001 |
| Drugs to treat infection | 2,624 (37%) | 7,289 (26%) | 2,784 (41%) | <0.001 |
| Other CNS drugs | 606 (8.5%) | 1,345 (4.8%) | 1,044 (15%) | <0.001 |
| Obs, gyn, and urinary tract drugs | 912 (13%) | 2,757 (9.8%) | 935 (14%) | <0.001 |
| Dermatology drugs | 2,012 (28%) | 5,559 (20%) | 2,003 (29%) | <0.001 |
| ENT drugs | 889 (12%) | 2,770 (9.8%) | 996 (15%) | <0.001 |
| Drugs used to treat eye conditions | 637 (8.9%) | 1,945 (6.9%) | 677 (9.9%) | <0.001 |
| Malignancy & immunosuppression drugs | 107 (1.5%) | 296 (1.1%) | 121 (1.8%) | <0.001 |
| Parkinson drugs | 127 (1.8%) | 149 (0.5%) | 72 (1.1%) | <0.001 |

* n (%)

† Pearson's Chi-squared test; Fisher's exact test

Supplementary Table 1- Number of patients in each cohort prescribed each drug one year before injury. Note: multiple patients were prescribed more than one drug. Drugs are grouped into categories which align with chapters of the BNF.

| **Characteristic** | N = 7,147* |
| --- | --- |
| Type of Burn |  |
| Burn - chemicals | 306 (4.3%) |
| Burn - electricity high | 27 (0.4%) |
| Burn - electricity low | 54 (0.8%) |
| Burn - other | 88 (1.2%) |
| Burn - exposure heat | 69 (1.0%) |
| Burn - flame | 667 (9.3%) |
| Burn - hot liquid | 784 (11%) |
| Burn - hot object | 246 (3.4%) |
| Burn - steam | 35 (0.5%) |
| Unknown | 4,871 (68%) |
| TBSA |  |
| <20% | 2,825 (40%) |
| 20% to 49% | 117 (1.6%) |
| >50% | 70 (0.1%) |
| Unknown | 4,135 (58%) |
| Airway Burn |  |
| No | 2,230 (31%) |
| Uncertain | 36 (0.5%) |
| Yes | 36 (0.5%) |
| Unknown | 4,845 (68%) |
| Smoke Inhalation |  |
| No | 2,225 (31%) |
| Uncertain | 33 (0.4%) |
| Yes | 44 (0.6%) |
| Unknown | 4,845 (68%) |

*n (%)

Supplementary Table 2- Description of burn sustained by patients in the burns cohort. Large number of unknowns can be attributed to incompleteness in the COBIS database.

| Characteristic | IRR * | 95% CI * | P-value |
| --- | --- | --- | --- |
| TBSA |  |  |  |
| <20% | Reference | Reference |  |
| 20% to 49% | 2.39 | 1.49, 4.13 | <0.001 |
| >50% | 2.22 | 1.13, 5.21 | 0.038 |
| Smoke Inhalation |  |  |  |
| No | Reference | Reference |  |
| Uncertain | 1.99 | 0.96, 5.08 | 0.1 |
| Yes | 1.09 | 0.55, 2.56 | 0.8 |
| Age in years† | 1.02 | 1.01, 1.02 | <0.001 |
| Sex |  |  |  |
| Male | Reference | Reference |  |
| Female | 1.79 | 1.60, 2.00 | <0.001 |
| Ethnic group |  |  |  |
| Any White background | Reference | Reference |  |
| Any mixed ethnic group | 0.13 | 0.03, 0.82 | 0.013 |
| Any Asian background | 0.54 | 0.33, 0.94 | 0.021 |
| Any Black background | 0.25 | 0.08, 0.97 | 0.022 |
| Other ethnic group | 0.59 | 0.31, 1.25 | 0.13 |
| Unknown ethnicity | 0.43 | 0.37, 0.49 | <0.001 |
| SIMD Quintile |  |  |  |
| 1 | Reference | Reference |  |
| 2 | 0.87 | 0.75, 1.02 | 0.078 |
| 3 | 0.81 | 0.69, 0.95 | 0.01 |
| 4 | 0.62 | 0.53, 0.74 | <0.001 |
| 5 | 0.59 | 0.48, 0.71 | <0.001 |
| Pre-injury Opioid Use | 6.66 | 6.02, 7.38 | <0.001 |
| ICU | 1.34 | 1.09, 1.66 | 0.008 |
| Total Morbidity Count | 1.49 | 1.35, 1.65 | <0.001 |
| Multimorbidity | 1.81 | 1.42, 2.36 | <0.001 |
| Alcohol abuse | 1.57 | 1.25, 2.01 | <0.001 |
| Drug Abuse | 1.06 | 0.66, 1.81 | 0.8 |
| Depression | 1.63 | 1.03, 2.78 | 0.053 |
| Psychosis | 1.07 | 0.65, 1.94 | 0.8 |

*IRR = Incidence Rate Ratio, CI = Confidence Interval

†Calculated for each one year increase in age

Supplementary Table 3- Univariable analysis of factors associated with an increase in opioid prescriptions following discharge from burn admission. This table includes just the burn cohort,


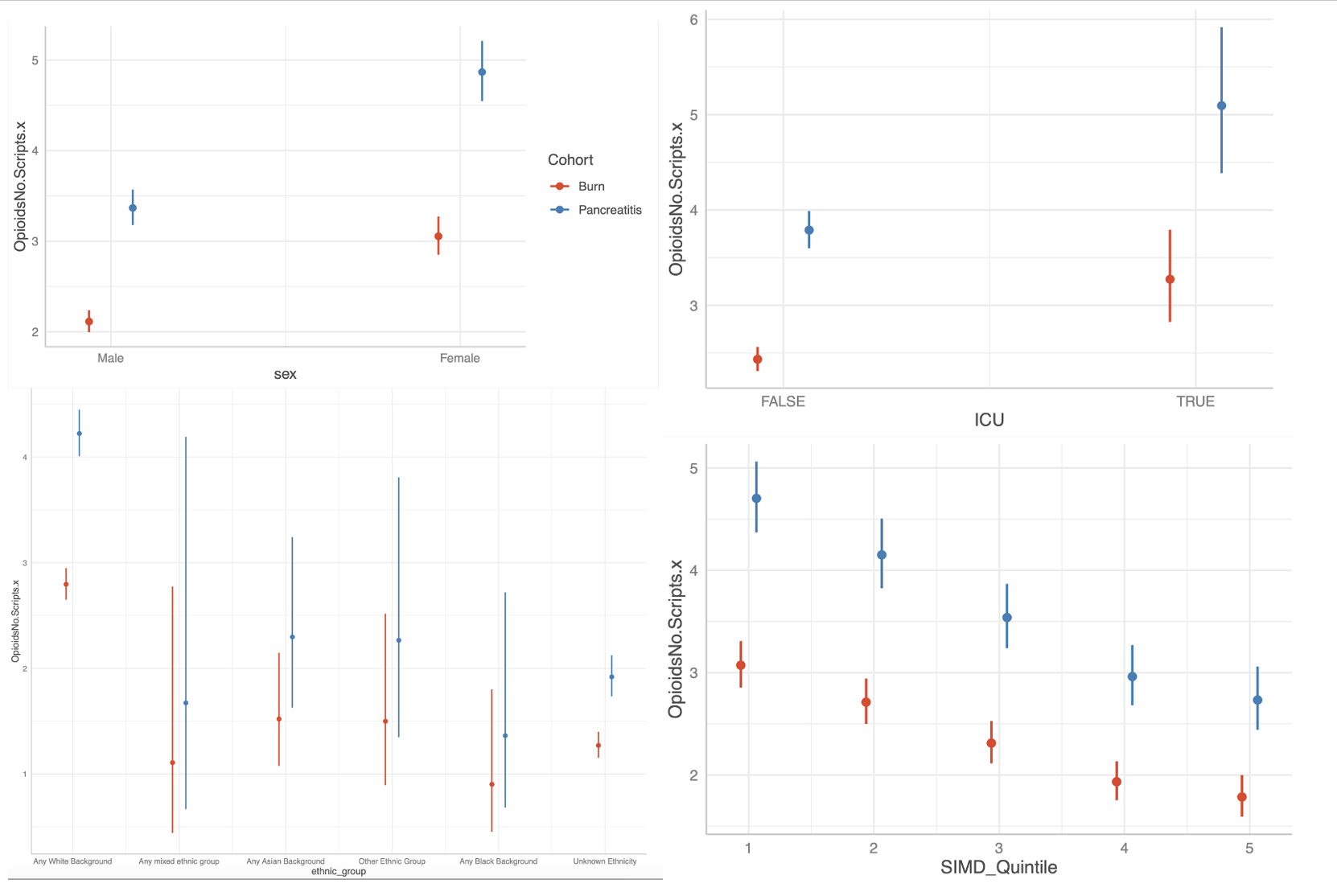

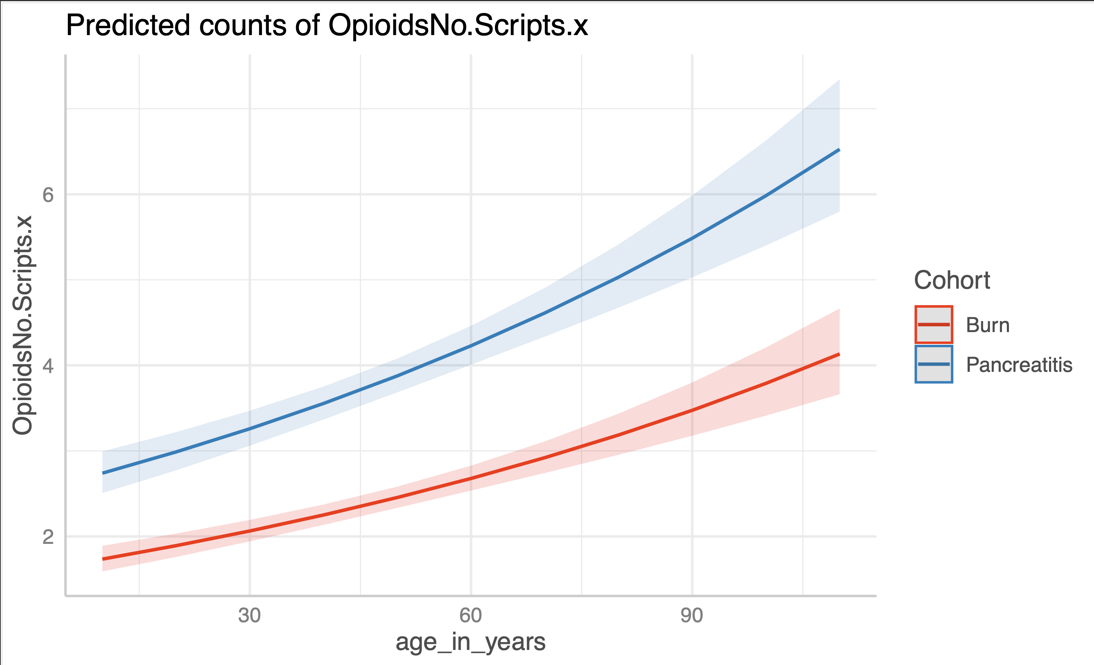

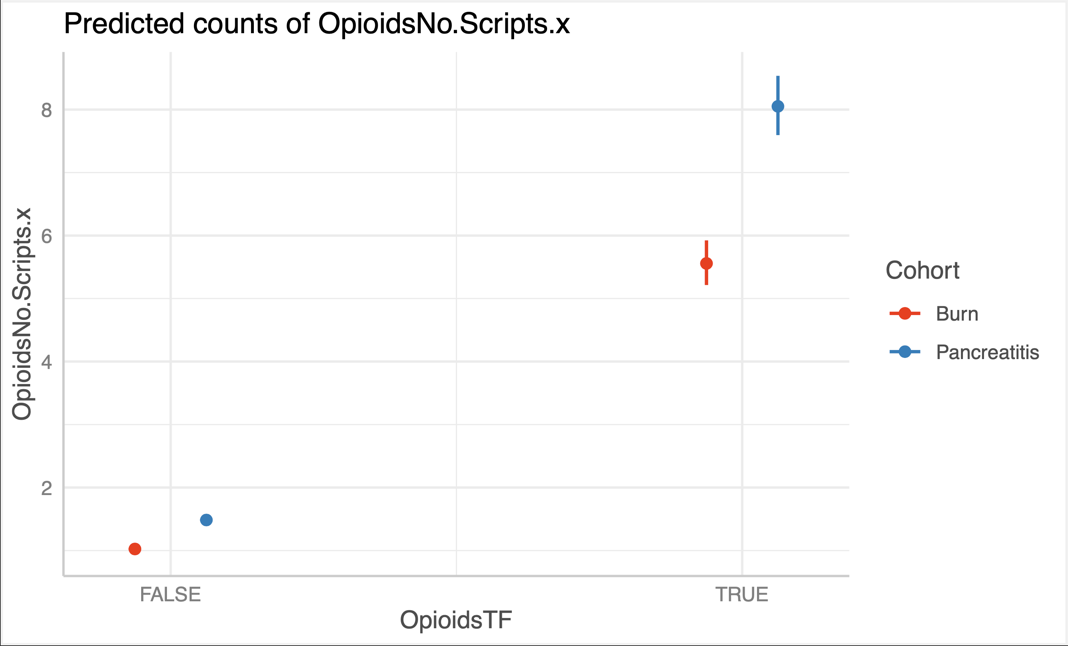


Male Female

Sex

False True

ICU

False True

Previous Opioid use

30 60 90

SIMD Quintile

White Mixed Asian Other Black Unknown

5

4

3

2

1

1

6

6

6

2

2

2

2

2

5

5

5

3

3

3

3

4

4

4

4

4

4

No. of opioid prescriptions post discharge

No. of opioid prescriptions post discharge

No. of opioid prescriptions post discharge

No. of opioid prescriptions post discharge

No. of opioid prescriptions post discharge

No. of opioid prescriptions post discharge

Ethnic Group

Age

Supplementary Figure 1- Graphical representation of number of opioid prescriptions post discharge when comparing different factors of interest using univariable analysis of both cohorts

| **Factor** | IRR* | 95% CI* | p-value |
| --- | --- | --- | --- |
| Sex |  |  |  |
| Male | Reference | Reference |  |
| Female | 1.46 | 1.25, 1.70 | <0.001 |
| SIMD Quintile |  |  |  |
| 1 | Reference | Reference |  |
| 2 | 0.85 | 0.70, 1.05 | 0.13 |
| 3 | 0.84 | 0.67, 1.04 | 0.11 |
| 4 | 0.83 | 0.66, 1.04 | 0.1 |
| 5 | 0.59 | 0.45, 0.77 | <0.001 |
| Age in years† | 1.01 | 1.01, 1.02 | <0.001 |
| Pre Injury Opioid use | 5.75 | 4.88, 6.81 | <0.001 |
| ICU | 2.04 | 1.54, 2.75 | <0.001 |
| TBSABOTH |  |  |  |
| <20% | Reference | Reference |  |
| 20% to 49% | 3.18 | 2.05, 5.15 | <0.001 |
| >50% | 1.77 | 0.98, 3.57 | 0.078 |

* IRR = Incidence Rate Ratio, CI = Confidence Interval

†Calculated for each one year increase in age

Supplementary Table 4- Subgroup multivariable analysis of factors associated with an increase in opioid prescriptions following discharge from a burn injury. This table includes just the burns cohort.
